# Antioxidant and Antihyperglycemic Properties of Three Banana Cultivars (*Musa* spp.)

**DOI:** 10.1155/2016/8391398

**Published:** 2016-10-30

**Authors:** Bukola C. Adedayo, Ganiyu Oboh, Sunday I. Oyeleye, Tosin A. Olasehinde

**Affiliations:** ^1^Functional Foods and Nutraceuticals Unit, Department of Biochemistry, Federal University of Technology, PMB 704, Akure 340001, Nigeria; ^2^Department of Biomedical Technology, Federal University of Technology, PMB 704, Akure 340001, Nigeria; ^3^Nutrition and Toxicology Division, Food Technology Department, Federal Institute of Industrial Research, Oshodi, PMB 21023, Lagos 10001, Nigeria

## Abstract

*Background*. This study sought to investigate the antioxidant and antihyperglycemic properties of* Musa sapientum* (Latundan banana) (MSL),* Musa acuminata* (Cavendish banana) (MAC), and* Musa acuminate* (Red Dacca) (MAR).* Materials and Methods.* The sugar, starch, amylose, and amylopectin contents and glycemic index (GI) of the three banana cultivars were determined. Furthermore, total phenol and vitamin C contents and *α*-amylase and *α*-glucosidase inhibitory effects of banana samples were also determined.* Results*. MAC and MAR had the highest starch, amylose, and amylopectin contents and estimated glycemic index (eGI) with no significant different while MSL had the lowest. Furthermore, MAR (1.07 mg GAE/g) had a higher total phenol content than MAC (0.94 mg GAE/g) and MSL (0.96 mg GAE/g), while there was no significant difference in the vitamin C content. Furthermore, MAR had the highest *α*-amylase (IC_50_ = 3.95 mg/mL) inhibitory activity while MAC had the least (IC_50_ = 4.27 mg/mL). Moreover, MAC and MAR inhibited glucosidase activity better than MSL (IC_50_ 3.47 mg/mL).* Conclusion*. The low sugar, GI, amylose, and amylopectin contents of the three banana cultivars as well as their *α*-amylase and *α*-glucosidase inhibitory activities could be possible mechanisms and justification for their recommendation in the management of type-2 diabetes.

## 1. Introduction

Previous experimental investigations involving the treatment and/or management of diabetes have revealed that decrease in blood glucose in diabetic patients with hyperglycemia can reduce diabetic consequences and prevent risk of diabetic complications such as hypertension [1]. Many reports have shown diverse therapeutic and dietary strategies in the treatment and/or management of diabetes and its complications [2, 3]. Willett et al. [4] reported that consumption of slowly absorbed and low glycemic index carbohydrates can contribute to low blood glucose response in diabetic individuals. Furthermore, some epidemiological studies have revealed that replacing high glycemic index foods with low glycemic foods can reduce the risk of diabetes [5, 6]. Other reports have also revealed that inhibition of carbohydrate hydrolyzing enzymes (*α*-amylase and *α*-glucosidase) can retard the release of glucose into the blood stream thereby preventing hyperglycemia [7–9]. The use of dietary approach for the management of diabetes has gained a lot of interest recently. Natural inhibitors of *α*-amylase and *α*-glucosidase activity present in plant foods have little or no side effects and more advantages due to their biologically active constituents such as polyphenols. Augmenting endogenous antioxidants via consumption of plant-based antioxidant compounds such as polyphenols can prevent oxidative stress, another culprit in pathophysiology of type 2 diabetes which is a major risk of diabetic complications [10, 11].

Banana is one of the most consumed fruits in tropical and subtropical regions of the world [12]. In Nigeria some of the most common edible banana cultivars include* Musa sapientum* (Latundan banana) (MSL) also referred to as* Musa paradisiaca*,* Musa acuminata* (Cavendish banana) (MAC), and* Musa acuminate* (Red Dacca) (MAR). Kanazawa and Sakakibara [13] reported the antioxidant properties and radical scavenging activities of some banana species. There are also some reports on the glycemic index and antihyperglycemic and antidiabetic properties of* Musa paradisiaca* [14, 15]. However, to the best of our knowledge, there is dearth of information on the holistic and comparative studies on the antioxidant and antihyperglycemic properties of some banana cultivars (*Musa* spp.). This present study aims to determine the sugar, starch, amylose and amylopectin contents, and estimated glycemic index (eGI) as well as total phenol and vitamin C contents of three banana [*Musa sapientum* (Latundan banana) (MSL),* Musa acuminata* (Cavendish banana) (MAC), and* Musa acuminate* (Red Dacca) (MAR)] cultivars. The antioxidant properties and inhibitory effects of the banana cultivars on carbohydrate trimming enzymes (*α*-amylase and *α*-glucosidase activities) were also determined.

## 2. Materials and Methods

### 2.1. Sample Collection

Three different varieties of matured ripe banana fruits,* Musa sapientum* (*Musa paradisiaca*),* Musa acuminata* (Red Dacca), and* Musa acuminata* (Cavendish banana), were obtained from a local market in Akure, Ondo State, Nigeria. Authentication of the samples was carried out at the Department of Crop Soil and Pest (CSP), Federal University of Technology, Akure, Nigeria.

### 2.2. Chemicals and Reagents

Gallic acid and Folin-Ciocalteu's reagent were procured from Sigma-Aldrich (St. Louis, MO), while DPPH was from Sigma-Aldrich Chemie (Steinheim, Germany). Unless stated otherwise, all the chemicals and reagents used were of analytical grades and the water was glass distilled.

### 2.3. Aqueous Sample Preparation

The edible portion (pulp) was separated from the peels. The pulp was spliced and oven dried at 50°C and thereafter milled into powdery form. Each sample (10 g) was soaked in 100 mL distilled water for 16 h on an orbital shaker [16]. The mixture was filtered using Whatman filter (number 2) and later centrifuged at 400 ×g for 10 min to obtain a clear supernatant which was used for vitamin C and total phenol contents as well as *α*-amylase and *α*-glucosidase activities inhibitory assays. Dried powder sample was kept for starch, sugar, and amylose contents and eGI analyses.

### 2.4. Starch and Sugar Determination

Starch and sugar were extracted from 0.02 g of the pulverized sample using 80% hot ethanol. The mixture was then centrifuged at 2000 rpm for 10 min after which the supernatant was decanted and used for free sugar analysis, while the residue was used for starch analysis [17]. For sugar analysis, 0.2 mL of the diluted supernatant was mixed with 0.5 mL of phenol solution (5%) and 2.5 mL of H_2_SO_4_ (absolute). The mixture was allowed to cool to room temperature before reading the absorbance at 490 nm. The residue was hydrolyzed with 7.5 mL of perchloric acid for 1 hr, diluted to 25 mL with distilled water, and filtered through Whatman filter paper (number 2). Then 0.05 mL of the filtrate was mixed with 0.5 mL of phenol solution (5%) and 2.5 mL H_2_SO_4_ (absolute). The mixture was allowed to cool to room temperature and the absorbance was read at 490 nm. Starch and total free sugar contents of the sample were calculated from a glucose standard curve prepared along with the sample.

### 2.5. Amylose and Amylopectin Content Determination

The samples (0.1 g) were mixed with 1 mL of 95% ethanol and 9.2 mL of 1 N NaOH and heated at 100°C in a water bath for 10 min. After cooling, 0.5 mL of diluted sample was mixed with 0.1 mL of 1 N acetic acid solution and 0.2 mL of iodine solution (0.2% I_2_ in 2% KI). The test mixture was made up to 10 mL with distilled water, mixed, and left for 20 min for color development. Thereafter, the absorbance was read at 620 nm and amylase content was calculated using standard amylase. Amylopectin was calculated using the following formula: amylopectin = starch value − amylose value [18].

### 2.6. Determination of Estimated Glycemic Index

The sample (25 mg) was weighed into a beaker; thereafter, 1 mg of pepsin in 10 mL HCl + KCl buffer (pH 1.5) was added and then incubated at 40°C for 60 min in a shaking water bath. The digest was then diluted with phosphate buffer pH 6.9 before the addition of 2.5 mL *α*-amylase solution and incubated at 37°C. 200 *μ*L of the digest was taken into test tube at 30 min interval (0, 30, 60, 90, 120, 150, and 180 min). The aliquots were boiled for 15 min before addition of 500 *μ*L sodium acetate pH 4.75 followed by 5 *μ*L of *α*-glucosidase solution and then incubated for 45 min at 60°C. 200 *μ*L DNSA solution was added and incubated for 5 min at 100°C followed by addition of 2 mL distilled water and then centrifuge at 3000 rpm for 5 min. The supernatant was decanted and the absorbance was read at 540 nm. The sum of areas under curve for each sample was divided by the sum of areas under curve for standard glucose and multiplied by 100. The value obtained is the glycemic index [19].

### 2.7. Determination of Total Phenol Content

The total phenol content was determined according to the method of Singleton et al. [20]. Briefly, appropriate dilutions of the banana samples were oxidized with 2.5 mL 10% Folin-Ciocalteu's reagent (v/v) and neutralized by 2.0 mL of 7.5% sodium carbonate. The reaction mixture was incubated for 40 min at 45°C and the absorbance was measured at 765 nm in the spectrophotometer. The total phenol content was subsequently calculated as gallic acid equivalent.

### 2.8. Determination of Vitamin C Content

The vitamin C content of the samples was determined according to the method used by Ademiluyi et al. [21]. Briefly 5 g of the samples was sampled by 100 mL H_2_O, and 10 mL of the sample was mixed with 25 mL of glacial acetic acid and titrated against standardized 2,6-dichloroindophenol (0.05 g/100 mL) solution.

### 2.9. Determination of ABTS^*∗*^ Scavenging Ability

The total antioxidant capacity was determined based on 2,2-azinobis 3-ethylbenzothiazoline 6-sulfonate radical (ABTS^*∗*^) scavenging ability of the sample according to the method described by Re et al. [22]. ABTS radical was generated by reacting ABTS aqueous solution (7 mM) with K_2_S_2_O_8_ (2.45 mM, final concentration) in the dark for 16 h and adjusting the absorbance at 734 nm to 0.700 with ethanol. Appropriate dilution of the samples (0.2 mL) was added to 2.0 mL ABTS radical solution and the absorbance was measured at 734 nm after 15 min. The trolox equivalent antioxidant capacity (TEAC) was subsequently calculated using trolox as the standard.

### 2.10. Free Radical Scavenging Assay

**Figure 1:**
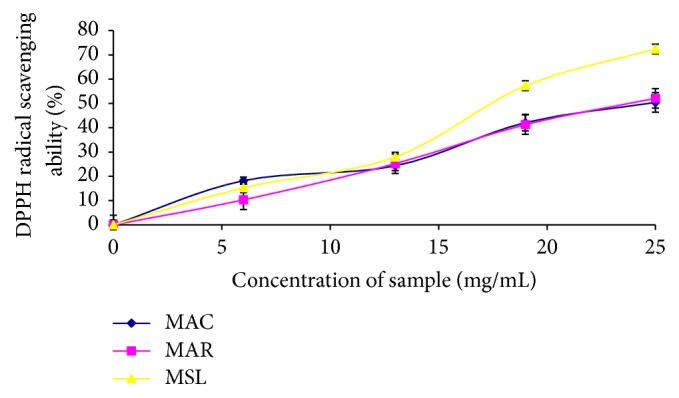
DPPH radical scavenging ability of* Musa sapientum* (Latundan banana) (MSL),* Musa acuminata* (Cavendish banana) (MAC), and* Musa acuminate* (Red Dacca) (MAR).

The free radical scavenging ability of the sample against DPPH radical was evaluated as described by Gyamfi et al. [23]. Briefly, an appropriate dilution of the samples (1 mL) was mixed with 1 mL 0.4 mM DPPH radicals in methanolic solution. The mixture was left in the dark for 30 min, and the absorbance was taken at 516 nm. The control was carried out by using 2 mL DPPH solution without the test samples. The DPPH radical scavenging ability was subsequently calculated as percentage control (Figure 1).

### 2.11. *α*-Amylase Inhibition Assay

**Figure 2:**
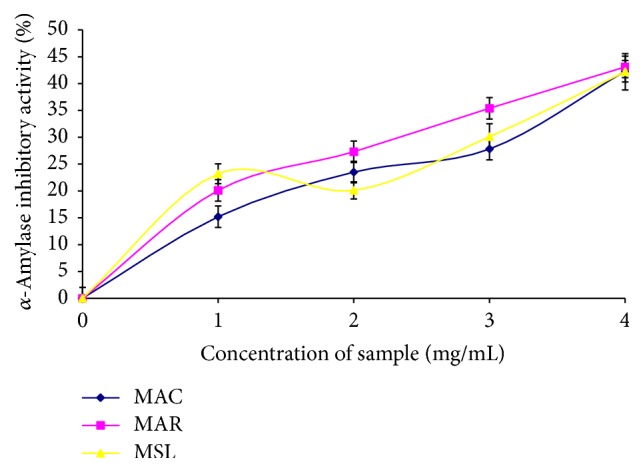
*α*-Amylase inhibitory activity of* Musa sapientum* (Latundan banana) (MSL),* Musa acuminata* (Cavendish banana) (MAC), and* Musa acuminate* (Red Dacca) (MAR).

The aqueous samples (500 *μ*L) and 500 *μ*L of 0.02 M of sodium phosphate buffer (pH 6.9 with 0.006 mol·L^−1^ NaCl) containing hog pancreatic *α*-amylase (EC 3.2.1.1; 0.5 mg/mL) were incubated at 25°C for 10 min. Then, 500 *μ*L of 1% starch solution in 0.02 M sodium phosphate buffer (pH 6.9 with 0.006 M NaCl) was added to the reaction mixture. Thereafter, the reaction mixture was incubated at 25°C for 10 min and stopped with 1.0 mL of dinitrosalicylic acid (DNSA). The mixture was then incubated in boiling water for 5 min and cooled to room temperature. The reaction mixture was then diluted by adding 10 mL of distilled water, and absorbance was measured at 540 nm in a UV-Visible spectrophotometer (Model 6305; Jenway, Barloworld Scientific, Dunmow, United Kingdom) [24] (Figure 2).

### 2.12. *α*-Glucosidase Inhibition Assay

**Figure 3:**
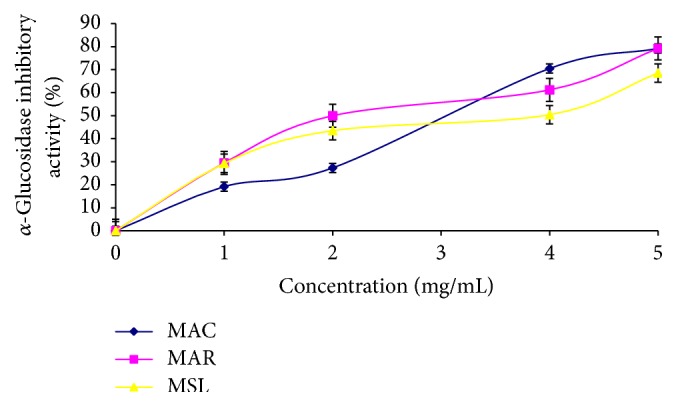
*α*-Glucosidase inhibitory activity of* Musa sapientum* (Latundan banana) (MSL),* Musa acuminata* (Cavendish banana) (MAC), and* Musa acuminate* (Red Dacca) (MAR).

Aqueous sample (50 *μ*L) and 100 *μ*L of *α*-glucosidase solution (1.0 U/mL) were incubated at 25°C for 10 min. Thereafter, 50 *μ*L of 5 M* p*-nitrophenyl-*α*-D-glucopyranoside solution in 0.1 mol·L^−1^ phosphate buffer (pH 6.9) was added. The reaction mixture was then incubated at 25°C for 5 min, and then absorbance was measured at 405 nm in the spectrophotometer. The *α*-glucosidase inhibitory activity was expressed as percentage inhibition [25] (Figure 3).

### 2.13. Data Analysis

The results of the three replicates were pooled and expressed as mean ± standard error (SE). Student's* t*-test, one-way analysis of variance (ANOVA), and the least significance difference (LSD) were carried out [26]. Significance was accepted at *P* ≤ 0.05. EC_50_ was determined using linear regression analysis.

## 3. Findings

Table 1 shows the carbohydrate content and glycemic index of three banana varieties. MSL (51.36 mg/g) had higher starch content than other varieties; however, there was no significant (*P* > 0.05) difference in the starch content of MAC (57.31 mg/g) and MAR (58.13 mg/g). MAC (17.87 mg/g) had lower sugar content than MAR (19.50 mg/g) and MSL (18.52 mg/g). The amylose content of MAC (9.67 mg/g) and MSL (10.18 mg/g) was not significantly (*P* > 0.05) different but lower than amylose content of MAR (12.84 mg/g). Moreover, MSL had lower amylopectin content than MAC and MAR. The amylose/amylopectin ratio of the banana samples ranges from 0.20 to 0.33. The glycemic index of banana varieties as shown in Table 1 indicates that MSL (41.33%) had lower glycemic index compared to other varieties. There was no significant difference in the glycemic index between MAC (45.49%) and MAR (44.95%) varieties.

The total phenol content of MAC, MAR, and MSL was 0.94 mg GAE/g, 1.07 mg GAE/g, and 0.96 mg GAE/g, respectively (Table 2). The results also revealed that there was no significant difference in the vitamin C contents and ABTS radical scavenging activity of the three banana varieties (Table 2). Furthermore, the banana varieties scavenged DPPH radical in a dose dependent manner. However, MAR (IC_50_ = 18.09 mg/mL) and MSL (IC_50_ = 16.33 mg/mL) varieties had higher radical scavenging activity than MAC (IC_50_ = 21.84 mg/mL). The samples also inhibited *α*-amylase and *α*-glucosidase activities in a dose dependent manner, but MAR (IC_50_ = 3.95 mg/mL) and MSL (IC_50_ = 4.09 mg/mL) had the highest *α*-amylase activity compared to MAC (IC_50_ = 4.27 mg/mL). The result on *α*-glucosidase activity of the samples revealed that MAC (IC_50_ = 3.09 mg/mL) and MAR (IC_50_ = 3.02 mg/mL) had the highest *α*-glucosidase activity with no significant difference. However, MSL (IC_50_ = 3.47 mg/mL) had the least *α*-glucosidase activity amongst the samples. Furthermore, the Pearson correlation coefficient (Table 3) revealed that there was significant correlation (*P* = 0.01) between the phytoconstituents (total phenol, total flavonoid, and vitamin C contents) and the enzyme inhibition as well as antioxidant properties (DPPH and ABTS radical scavenging abilities) of the cultivars.

## 4. Discussion

Previous studies have shown that control of blood sugar and variations in glycemic index of different foods may be helpful in the management of type 2 diabetes [4, 27]. Consumption of starch-rich foods can increase postprandial blood glucose and insulin response [28]. In this study, the starch content of the banana varieties was moderately higher than that of some tropical fruits but lower than that of breadfruit reported by Oboh et al. [29]. However MSL banana had the lowest starch content compared to MAC and MAR. Consumption of lower starchy foods may contribute to decrease in glucose response; therefore, it could be an important factor in the management of diabetes. Hoover-Plow et al. [30] reported that low levels of starch content in apple and banana contributed to low blood glucose response in some selected diabetic patients. Furthermore, the observed low sugar content of the banana varieties could be beneficial especially to diabetic patients due to the fact that high consumption of sugars can increase the risk of type 2 diabetes and its complications such as cardiovascular diseases [31].

Amylose/amylopectin ratio is one of the major factors that influence blood glucose response and/or GI of food [32]. This is due to the fact that diets rich in amylose with low amylopectin content can induce low glycemic blood and insulin response [33]. Our findings revealed that the studied banana cultivars had low amylose and high amylopectin contents. The implication of this result is that starches with open branch structure that consist of large proportions of amylopectin than amylose are susceptible to enzymatic attack which can lead to rapid release of glucose into the bloodstream and hyperglycemia [28, 34]. However, despite the high amylopectin content, the banana cultivars displayed low glycemic response as revealed by their GI values.

Glycemic index is usually classified into low GI (0–55), medium GI (56–69), and high GI (≥70) using glucose as reference [29]. Our results revealed that MSL had lower GI than MAC and MAR. The low GI value exhibited by MSL cultivar could be attributed to its lower amylose and amylopectin contents compared to other studied cultivars. The high amylose in the MSL cultivar could slow down the digestion rate as a result of highly branched amylose structure, thereby limiting the rate at which glucose is released into the blood. This is contrary to the low amylose content which was observed in MAR and MAC samples. Therefore, consumption of MSL appears to be the best in the management of diabetes as it will reduce hyperglycemia and reduce the risk of diabetic complications especially cardiovascular diseases.

Chronic hyperglycemia has been implicated in the early onset and pathogenesis of diabetes and its complication [35]. There are indications that chronic hyperglycemia can induce the production of reactive oxygen species and ultimately lead to oxidative stress and pancreatic cell damage [3, 35]. The three banana cultivars exhibited radical scavenging activities as typified by their ABTS and DPPH radical scavenging abilities (Table 1). The result, therefore, agrees with the findings of Shian et al. [14] who reported that banana fruits have antioxidant properties. The radical scavenging activity of the banana samples has strong correlation with the vitamin C content but low correlation with phenolic contents (Table 3). Moreover previous finding has revealed that vitamin C is a strong antioxidant molecule and scavenger of oxygen-derived radicals such as hydroxyl (OH) radical and singlet oxygen [36], although the antioxidant properties of many plants alongside with their radical scavenging ability have been linked to their phenolic content [37, 38].

Rapid degradation of starchy foods by carbohydrate-hydrolyzing enzymes (*α*-amylase and *α*-glucosidase) leads to hyperglycemia [3]. Most common therapeutic approach to the treatment and management of diabetes involves the use of synthetic inhibitors of *α*-amylase and *α*-glucosidase activities to retard the release of glucose into the blood stream [39]. However, the use of these synthetic inhibitors such as acarbose and voglibose causes some side effects such as abdominal cramps, flatulence, and meteorism [40]. Dietary approach to the management of diabetes using plant with natural inhibitors of *α*-amylase and *α*-glucosidase has a greater advantage. Our findings revealed that the three banana cultivars inhibited *α*-amylase and *α*-glucosidase activities in a dose dependent manner. MAR cultivar had a higher inhibitory effect on *α*-glucosidase than *α*-amylase. Our findings revealed that there is a correlation between total phenol content and *α*-amylase and *α*-glucosidase inhibitory activities of the banana cultivars. A strong correlation was observed between total phenol content and *α*-glucosidase inhibition compared to amylase inhibition. This result correlates with the findings of [41] which reported a higher glucosidase activity of phenolic samples from soybean than its corresponding amylase activity. Moreover higher inhibition of *α*-glucosidase activity than its corresponding *α*-amylase is of great pharmaceutical importance in ameliorating the side effects induced by excess inhibition of *α*-amylase [9].

## 5. Conclusion

The knowledge of dietary fruits and vegetables to control and reduce hyperglycemia is essential in the treatment of diabetes. This is because dietary management is crucial to controlling spikes in blood glucose levels. In this study, the banana (MSL, MAR, and MAC) cultivars exhibited low GI index and antioxidant activities as typified by their radical scavenging abilities. The samples also had inhibitory effect on *α*-amylase and *α*-glucosidase activities. These abilities could be attributed to their phytochemicals which therefore justify their recommendations for the management of diabetes.

## Figures and Tables

**Table 1:** The starch, sugar, amylose and amylopectin contents, amylose/amylopectin ratio, and glycemic index (%) of *Musa sapientum* (Latundan banana) (MSL), *Musa acuminata* (Cavendish banana) (MAC), and *Musa acuminate* (Red Dacca) (MAR).

Parameter	MAC	MAR	MSL
Starch (g/100 g)	57.31 ± 2.62^b^	58.13 ± 2.13^b^	51.36 ± 1.8^a^
Sugar (g/100 g)	17.87 ± 0.42^a^	19.50 ± 0.52^b^	18.52 ± 0.45^b^
Amylose content (A) (g/100 g)	9.67 ± 0.42^a^	10.18 ± 0.45^a^	12.84 ± 0.63^b^
Amylopectin content (Am) (g/100 g)	47.64 ± 1.25^b^	47.95 ± 1.67^b^	38.52 ± 1.12^a^
GI (%)	45.49 ± 1.25^a^	44.95 ± 1.33^a^	41.33 ± 0.95^b^
A/Am	0.20	0.23	0.33

Value represents mean ± standard deviation of triplicate readings. Values with the same superscript letter on the same row are not significantly different (*P* > 0.05).

**Table 2:** Total phenol and flavonoid content and EC_50_ values of DPPH radical scavenging ability and inhibition of *α*-amylase and *α*-glucosidase activities of *Musa sapientum* (Latundan banana) (MSL), *Musa acuminata* (Cavendish banana) (MAC), and *Musa acuminate* (Red Dacca) (MAR).

Samples	MAC	MAR	MSL	Acarbose
Total phenol (mg GAE/g)	0.94 ± 0.01^a^	1.07 ± 0.02^b^	0.96 ± 0.03^a^	
Vitamin C (mg/g)	1.01 ± 0.03^a^	0.95 ± 0.03^a^	0.96 ± 0.02^a^	
ABTS (mmol TEAC/g)	5.03 ± 0.08^a^	4.98 ± 0.11^a^	5.01 ± 0.04^a^	
DPPH (mg/mL)	21.84 ± 1.25^b^	18.09 ± 0.98^a^	16.33 ± 1.05^a^	
*α*-Amylase (mg/mL)	4.27 ± 0.13^c^	3.95 ± 0.10^a^	4.09 ± 0.05^b^	0.18 ± 0.02^d^
*α*-Glucosidase (mg/mL)	3.09 ± 0.15^a^	3.02 ± 0.12^a^	3.47 ± 0.10^b^	0.22 ± 0.01^c^

Values represent mean ± standard deviation of triplicate readings. Values with the same superscript letter on the same row are not significantly different (*P* > 0.05).

**Table 3:** Pearson correlation coefficients for total phenol, vitamin C contents, amylase and glucosidase inhibitory, and free radical (DPPH and ABTS) scavenging abilities of the banana cultivars.

	*α*-Amylase	*α*-Glucosidase	DPPH radical	ABTS radical
			Scavenging ability	Scavenging ability
TP	0.283	0.590	0.207	0.032
VC	0.829	0.952	0.916	0.976

All correlation coefficients are significant at *P* < 0.01 (two-tailed).

TP = total phenol; VC = vitamin C.
